# Left hippocampus–amygdala complex macro- and microstructural variation is associated with BDNF plasma levels in healthy elderly individuals

**DOI:** 10.1002/brb3.334

**Published:** 2015-05-26

**Authors:** Antonietta Manna, Fabrizio Piras, Carlo Caltagirone, Paola Bossù, Stefano L Sensi, Gianfranco Spalletta

**Affiliations:** 1 Molecular Neurology Unit, Center of Excellence on Aging (CeSI)Chieti, Italy; 2 Clinical and Behavioral Neurology, IRCCS Fondazione Santa LuciaRome, Italy; 3 Department of Neuroscience, “Tor Vergata” UniversityRome, Italy; 4 Department of Neuroscience and Imaging, ‘G. d'Annunzio’ UniversityChieti, Italy; 5 Institute for Memory Impairments and Neurological Disorders, University of California-IrvineIrvine, California; 6 Menninger Department of Psychiatry and Behavioral Sciences, Baylor College of MedicineHouston, Texas

**Keywords:** BDNF, brain volume, DTI

## Abstract

**Introduction:**

Deep brain gray matter (GM) structures are involved in several neurodegenerative disorders and are affected by aging. In this study, we investigated the potential relationship between levels of brain-derived neurotrophic factor (BDNF), a putative biomarker of age- and clinically relevant brain dysfunctions, and the presence of structural modifications that were evaluated by magnetic resonance imaging in six deep GM structures.

**Methods:**

Volume changes and diffusion tensor imaging (DTI) scalars were studied in the thalamus, putamen, hippocampus, caudate nucleus, amygdala and pallidum of a cohort of 120 healthy subjects. The cohort included young (18–39 years old), adult (40–59 years old) and elderly (60–76 years old) subjects.

**Results:**

No correlations were seen in the young and adult cohorts. In the elderly group, we observed reduced BDNF levels that correlated with increased DTI-based mean diffusivity occurring in the left hippocampus along with decreased normalized volume in the left amygdala.

**Conclusions:**

These findings suggest that, in elderly subjects, BDNF may exert regional and lateralized effects that allow the integrity of two strategic deep GM areas such as the hippocampus and the amygdala.

## Introduction

Changes in deep gray matter (GM) structures, such as the hippocampus and amygdala, have often been associated with a variety of behavioral modifications. As these structures are greatly affected by neurodegeneration and a major target for aging-related processes (Harman 1991; Farooqui and Farooqui 2009), a better understanding of how physiological aging facilitates the neurodegeneration of these structures is crucial to maximize the efficacy of preventive and rehabilitative measures.

When studying brain changes that occur with aging or neurodegenerative processes, it is crucial to choose the right neuroimaging approach in order to gain the maximum amount of information from scans. For example, high-resolution T1-weighted MR images provide anatomical detail at the macroscopic level, thereby allowing an effective investigation of age-related volume shrinkage processes occurring in cortical and subcortical GM structures (Pfefferbaum et al. 1994; Good et al. 2001; Fox and Schott 2004). The same methodological approach can also help to characterize patterns and rate of progression of aging-driven brain atrophy. Diffusion tensor imaging (DTI) is indicated in the study of microarchitecture variations and for the investigation, with cellular level inference, of early pathological alterations. The technique provides important physiological information than cannot otherwise be obtained by employing conventional MRI (Basser 1995; Le Bihan 1995; Basser and Pierpaoli 1996). Quantification of water diffusion in brain tissues with DTI is indeed a powerful procedure that allows the analysis of white matter (WM) organization and integrity (Pierpaoli et al. 1996). In contrast, since GM is less organized in orientation than WM, DTI, at least in the cerebral cortex, becomes less effective when aiming at analyzing GM structures. However, thanks to the proximity of coherent WM, deep GM structures can be evaluated with DTI as these structures exhibit high directionality in diffusion (Ziyan and Westin 2008). Among DTI parameters, mean diffusivity (MD) is a quantitative measure of directionally averaged diffusion and has been successfully employed to study microstructural alterations of deep GM regions (Müller et al. 2007; Cherubini et al. 2009; Piras et al. 2011). Increased MD in the GM is thought to be linked to enlargement of the extracellular space. MD is therefore a good indicator of the cytoarchitectural damage that occurs along with neurodegeneration (Sykova 2004; Kantarci et al. 2005). In deep GM structures, these changes may reflect either direct damage or secondary reactive degeneration following the disruption of connecting WM tracts (O'Sullivan et al., 2004).

In physiological conditions, extracellular water diffusion is influenced by different factors such as the size of the pores present between cells, integrity of cellular and axonal structures, and variations in the cell density and surface (Sykova and Nicholson, 2008; Le Bihan, 2007). These structural components affect the efficacy of synaptic and or extrasynaptic transmission (Sykova 2004) and have a great impact on the overall strength of synaptic functioning and, ultimately, cognitive functions (O'Sullivan et al., 2004; Cherubini et al. 2009; Piras et al. 2011). Increased MD values and volume shrinkage are thought to be the biological underpinnings of the damage of neuronal somata and/or the neuronal loss that occurs in aging and/or neurodegeneration (Basser 1995).

Thus, the primary difference between volume and diffusivity indices is that volume shrinkage reflects macroscopic structural alterations while diffusivity parameters are more indicative of structural variations occurring at cellular and molecular levels (Le Bihan 1995). These differences highlight the importance of measuring both parameters when one wants to carefully evaluate alterations taking place in deep GM structures. Although combined evaluation with different MR techniques has produced a considerable amount of information about microstructural alterations and atrophy of deep GM nuclei (Cherubini et al. 2009), previous studies have also often produced inconsistent and discordant results (Good et al. 2001; Liu et al. 2003; Szentkuti et al. 2004; Lemaître et al. 2005; Walhovd et al. 2005) and, in most cases, missed the subregional localization of the atrophic processes.

In many studies, the neurotrophin brain-derived neurotrophic factor (BDNF) has been indicated as a putative biomarker for a variety of brain dysfunctions. BDNF and its receptor tyrosine kinase (TrkB) are highly concentrated in the hippocampus (Phillips et al. 1990; Wetmore et al. 1990; Murer et al. 2001), a key region involved in the modulation of cognition and memory (Kang and Schuman 1995; Figurov et al. 1996; Stoop and Poo 1996; Pang et al. 2004; Tanaka et al. 2008). Furthermore, BDNF is thought to contribute to neurogenesis taking place in the dentate gyrus (Takahashi et al. 1999; Benraiss et al. 2001; Pencea et al. 2001). In humans, high BDNF levels have been linked to enlargements of hippocampal volumes and enhanced spatial memory performances (Erickson et al. 2010, 2012). Furthermore, decreased BDNF, at the plasma and serum level, has been associated with behavioral and cognitive deficits observed in neurodegenerative and psychiatric disorders (Sen et al. 2008; Erickson et al. 2012). In line with this, Weinstein and colleagues recently found that higher serum levels of BDNF protect against future occurrence of dementia and AD (Weinstein et al. 2014). Of note, it should be underlined that BDNF is also produced outside the central nervous system (Scarisbrick et al. 1993; Timmusk et al. 1993) and the trophin is also stored and released from blood platelets and immune cells (Yamamoto and Gurney 1990; Kerschensteiner et al. 1999; Gielen et al. 2003). Given the central role exerted by BDNF in controlling neural plasticity and cognition, evaluation of the trophin levels may represent a viable indicator to assess age-related changes of neuronal well-being as well as a marker of pathological brain dysfunctions.

Despite growing evidence of a strong relationship occurring between changes in BDNF levels and corresponding variations of some brain structures, so far no studies have investigated how changes in the neurotrophin levels may affect or relate to structural alterations of deep GM regions. A recent study, performed on elderly subjects, measured volumes of deep GM regions along with BDNF serum levels and evaluated whether age-related reductions in BDNF were associated with GM volume loss and memory deficits (Erickson et al. 2010). Another study showed that, in the elderly, increased hippocampal size is associated with higher BDNF concentrations (Erickson et al. 2011). Finally, in elderly subjects who reported cognitive benefits from aerobic training, evaluation of neuronal activity with functional magnetic resonance imaging (fMRI) together with analysis of changes in several neurobiological markers (including BDNF) indicated that these markers positively relate to increased levels of brain plasticity (Voss et al. 2013). In summary, previous brain-aging studies successfully investigated micro- and macrostructural brain alterations alone or, alternatively, studied macrostructural changes in conjunction with variations of serum BDNF levels; however, so far, no study had evaluated changes simultaneously occurring at all the three levels.

This study is the first that attempts to overcome the limitations of previous investigations on effects of aging in subcortical GM structures. To this aim, in a hypothesis-free fashion, we explored changes in volume, DTI scalars along with evaluation of serum BDNF levels in six deep GM structures (i.e., thalamus, putamen, hippocampus, caudate nucleus, amygdala, and pallidum) in a cohort of healthy subjects divided into three age brackets: young (18–39 years old), middle aged (40–59 years old), and elderly (60–76 years old) subjects.

## Experimental Procedures

### Participants

We recruited 120 healthy subjects [47 males (39.1%); mean age ± SD = 40.1 ± 15.3 years, range 18–76; mean education ± SD = 14.6 ± 3.3 years, range 5–24] for the study. The only inclusion criterion was age between 18 and 80 years. Exclusion criteria encompassed: (1) suspicion of cognitive impairment or dementia based on a Mini-Mental State Examination [(MMSE) (Folstein et al. 1975)] score lower or equal to 26 consistent with normative data collected in the Italian population and confirmed by a detailed clinical neuropsychological evaluation using the mental deterioration battery (MDB) (Carlesimo et al. 1996) and clinical criteria for Alzheimer's dementia established by the National Institute on Aging and the Alzheimer's Association (McKhann et al. 2011), or Mild Cognitive Impairment (Petersen and Morris 2005), (2) subjective complaint of memory difficulties or of any other cognitive deficits regardless of whether or not these interfere with daily living activities, (3) vision and hearing loss that could potentially interfere with testing procedures, (4) major medical illnesses (i.e., unstable diabetes, obstructive pulmonary disease, or asthma; hematological and oncological disorders; pernicious anemia; significant gastrointestinal, renal, hepatic, endocrine, or cardiovascular system diseases; recently treated hypo-thyroidism), (5) current or reported psychiatric (assessed by the DSM-IV-TR SCID, First et al. 2002) or neurological (assessed by a clinical neurological evaluation) disorders (e.g., schizophrenia, mood disorders or anxiety disorders, stroke, Parkinson's disease, seizures, head injury with loss of consciousness, or any other significant mental or neurological condition), (6) known or suspected history of alcoholism or drug addiction and/or history of abuse, (7) any potential brain abnormality or microvascular lesion that was appearing with conventional FLAIR scans; in particular, the presence, severity, and location of vascular lesions were, in fact, computed according to the semiautomated method recently published by our group (Iorio et al. 2013). Finally, all subjects met the criteria for participation in an MRI study, and therefore had no previous head or neck surgery, no previous head trauma, no brain tumors and no metallic implants that could interfere with or cause injury due to the magnetic field. The cohort composition by age and gender is summarized in Table 1.

**Table 1:** Sociodemographic characteristics of 120 healthy subjects separated by age.

Age	18–39	40–59	60–76
Number	67	33	20
Gender male, *n* (%)	29 (43.2)	11 (33.3)	7 (35)
Years of Education, mean ± Standard Deviation	15.9 ± 2.6	14.0 ± 2.7	11.0 ± 3.6

The study was approved and undertaken in accordance with the guidelines of the Santa Lucia Foundation Ethics Committee. A written consent form was signed by all participants after they received a full explanation of the study procedures.

### Neuropsychological assessment

A neuropsychological test battery was only used to exclude subjects with dementia or cognitive impairment. To obtain a global index of cognitive impairment, we employed the Mini-Mental State examination MMSE (Folstein et al. 1975). The instrument is brief and easy to administer and is widely used to screen for cognitive deterioration. Subjects were also asked to perform the Multiple Features Targets Cancellation Task (MFTC, Gainotti et al. 2001), a test that assesses visuospatial explorative abilities and psychomotor processing speed. Moreover, we administered the Copy and Delayed Recall of Rey-Osterrieth's complex picture test (CROP and ROPR, respectively; Osterrieth 1944) to evaluate visual perception/constructional praxis, perceptual organizational skills, planning, and problem-solving. We also chose three tests from the mental deterioration battery (MDB, Carlesimo et al. 1996) to provide information about functioning of different cognitive domains such as verbal memory (MDB Rey's 15-word Immediate Recall [RIR] and Delayed Recall [RDR]), logical reasoning (MDB Raven's Progressive Matrices’ 47 [PM47]), language (MDB Phonological (PVF), and Semantic (SVF) Verbal Fluency). Finally, set-shifting or cognitive flexibility was assessed using the Modified Wisconsin Card Sorting Test (MWCST; Heaton et al. 1993).

### Image acquisition

All 120 participants underwent the same MR imaging protocol, which included acquisition of standard clinical sequences (Fluid Attenuated Inversion Recovery (FLAIR) and PD-T2-weighted), whole-brain T1-weighted, and diffusion-weighted scanning using a 3T Allegra MR imager (Siemens, Erlangen, Germany), equipped with a standard quadrature head coil. All planar sequences were acquired along the anterior/posterior commissure line. Particular care was taken to center the subject's head in the head coil and to restrain movements using cushions. Whole-brain T1-weighted images were acquired in the sagittal plane using a modified driven equilibrium Fourier transform (MDEFT) sequence (TE/TR = 2.4/7.92 ms, flip angle = 15°, voxel size = 1 × 1 × 1 mm^3^). The echo-planar imaging technique (spin-echo-planar imaging, TE/TR = 89/8500 ms, bandwidth = 2126 Hz/vx; matrix size = 128 × 128; 80 axial slices, voxel size = 1.8 × 1.8 × 1.8 mm^3^) was used to collect diffusion-weighted volumes, with 30 isotropically distributed orientations for the diffusion-sensitizing gradients at a *b*-value of 1000 s·mm^2^ and six *b* = 0 images. Scanning was repeated three times to increase the signal-to-noise ratio.

### Image processing

Whole-image processing was performed using the Oxford Centre for Functional MRI of the Brain (FMRIB)'s Software Library (FSL, http://www.fmrib.ox.ac.uk/fsl/), version 4.1.

The FMRIB's Integrated Registration and Segmentation Tool (FIRST), included in FSL, was used for segmentation and volumetric analysis of the anatomical T1-weighted images. FIRST is a semiautomated model-based subcortical segmentation/registration tool that employes a Bayesian approach. The shape/appearance models used in FIRST are constructed from manually segmented images provided by the Center for Morphometric Analysis (CMA), MGH, Boston, MA, USA. The manual labels are parameterized as surface meshes and modeled as a point distribution model in which the geometry and variation of the shape of the structure are submitted as priors. Deformable surfaces are used to automatically parameterize the volumetric labels in terms of meshes; the deformable surfaces are constrained to preserve vertex correspondence across the training data. Furthermore, normalized intensities along the surface normals are sampled and modeled. The shape and appearance model is based on multivariate Gaussian assumptions. Shape is then expressed as a mean with modes of variation (principal components). On the basis of the learned models, FIRST searches through linear combinations of shape modes of variation for the most probable shape instance given the intensity distribution in the T1-weighted image. Particularly useful for structures with a low contrast-to-noise ratio, this method of segmentation makes the FIRST tool the best for finding the optimal border and extent of the structures considered, modeling these structures as surfaces.

DTI data were corrected for image distortions induced by eddy currents and head motion by applying 3D full affine (mutual information cost function) alignment of each image to the mean no diffusion-weighted (b0) image. After these corrections, DTI data were averaged and concatenated into 31 (1 b0 + 30 b1000) volumes. A diffusion tensor model was fit at each voxel, generating fractional anisotropy (FA) and MD maps. The FA maps were useful for obtaining a better coregistration with T1-weighted images (because the spatial distribution of signal intensities was similar in both image modalities), whereas MD values were used as an index of microstructural integrity within the deep gray matter nuclei. The FA maps created were registered to the whole-brain volumes extracted from T1-weighted images using a full affine (correlation ratio cost function) alignment with nearest-neighbor resampling. The calculated transformation matrix was applied to the MD maps with identical resampling options.

For each subject and each hemisphere, the procedure was as follows: (1) the caudate (body), putamen, pallidum, thalamus (thalamic nuclei and pulvinar), hippocampus (dentate gyrus, cornus ammonis (CA1, CA2, CA3, CA4), presubiculum and subiculum), and amygdala (basolateral complex, centro-medial and cortical nuclei) were segmented; (2) The resulting region-of-interest (ROI) segmentation and the coregistered FA map were then superimposed on the original T1-weighted volumes; (3) the obtained images were visually assessed by two trained radiologists to exclude misregistration or erroneous ROI identification; (4) finally, the volumes of the above-mentioned ROIs were calculated. Before statistical analysis, individual volume values were multiplied by a normalization factor obtained with the SIENAX tool (Smith et al. 2001) from the corresponding T1-weighted image. These segmented ROIs defined the binary masks where mean values of MD were calculated for each individual and each hemisphere. Previous studies used this method successfully for image processing (Cherubini et al. 2009; Piras et al. 2010, 2011).

### Blood serum collection and analysis

Venous blood was drawn from all subjects after an overnight fast. Within two hours from collection, serum was obtained after centrifugation of clotted blood samples, distributed into aliquots and stored at −80 °C until further analysis.

BDNF was detected in serum samples by sandwich ELISA according to the manufacturer's instructions (DuoSet ELISA, R&D Systems, Minneapolis, MN, USA). Briefly, 96-well immunoassay plates were coated overnight with 100 *μ*L/well of mouse anti-human BDNF monoclonal antibody (working concentration 2 *μ*g/mL) at room temperature (RT). Plates were then washed and blocked with assay buffer (PBS/BSA 1%) for 1 hour. After further washing, 100 *μ*L/well of serum samples (diluted 1:40) and serial dilutions of the BDNF standard (ranging from 23.4 to 1500 pg/mL BDNF) were incubated for 2 hours at RT. Plates were washed and 100 *μ*L of biotinylated mouse anti-human BDNF monoclonal antibody (working concentration 25 ng/mL) were added to each well and incubated for two hours at RT. After washing, 100 *μ*L/well of a streptavidin-enzyme conjugate (diluted 1:200) was added and incubated for 20 min at RT. After further washing, 100 *μ*L/well of a substrate solution (Tetramethylbenzide, Sigma, Saint Louis, MO, USA) was added to the wells to initiate a reaction, which was stopped after 30 min by adding 100 *μ*L/well of a stop solution (HCl 1M). The amount of BDNF was determined immediately by measuring absorbance at 450 nm using a microplate reader. The standard curve demonstrated a direct relationship between optical density and BDNF concentration. BDNF content was quantified against the standard curve. The detection limit was <4 pg/mL. Measurements were performed in duplicate and expressed as pg/mL. No cross-reactivity with other related neurotrophins (NGF, GDNF, NT-3; NT-4) was reported.

Serum (rather than plasma) was examined for two main reasons: (1) to avoid confounding results due to BDNF contained in platelets and released by platelet activation; (2) because it is the most commonly employed method to investigate relationships between changes in blood growth factors and individual differences in neuropsychiatric, cognitive, and exercise-driven variations occurring in humans (e.g., see review Brunoni et al. 2008).

### Statistical Analysis

Briefly, preliminary correlation analyses were conducted with the aim of selecting the variables to include in the multiple regression analysis; then, to evaluate the relevance, relative contribution and rate of change of these parameters with BDNF, multivariate procedures were run, with the BDNF values entered as dependent variable and the anatomical measures or the neuropsychological scores that survived the *P* < 0.05 threshold in the univariate correlation analyses as independent variables (quantitative predictors).

In particular, data from all 120 subjects were included in the statistical investigation and analyses were run separately by three age brackets (*young*, *adult* and *elderly* subjects). To investigate the association between changes in BDNF and micro- and macrostructural variations of six deep GM structures mean MD and volume values were considered as regressors.

First, we calculated partial correlation coefficients (Pearson's *r*) between BDNF and each of these parameters in the single ROIs. The analysis was separated by age category to assess the relationship between BDNF and: (1) micro-(MD) and (2) macro-(volume) anatomical measures. To approximate the strength of the difference between group correlation values, Fisher's r-to-Z transformation was carried out to compare the magnitude of the partial correlations. Since a high number of correlation analyses can increase the risk of a type I error (thereby leading to erroneously inferring the presence of a significant correlation) if calculated individually, the relationship between BDNF levels, volumetry, and MD variables was further assessed by means of a stepwise multiple regression analysis, that is, a multivariate statistical model which permits studying relationships without inflating the risk of a type I error (Draper and Smith 1981). This method of regression is a data-driven automatic procedure for statistical model selection in cases in which there are a large number of potential explanatory variables and no underlying theory for the model selection. We chose to perform both forward and backward stepwise regressions by considering all independent variables jointly. The *forward selection* approach starts with no variables in the model, tests the addition of each variable using a chosen model comparison criterion (statistically significant variable), adds the variable (if any) that improves the model most, and repeats this process until adding another variable does not improve the model; inversely, the *backward elimination* technique starts with all candidate variables, tests the deletion of each variable using a chosen model comparison criterion, deletes the variable (if any) that improves the model most by being deleted, and repeats this process until no further improvement is possible (Derksen and Keselman 1992). Results that are found valid by both procedures (forward and backward) are eventually taken in account. Finally, because of the possible multicollinearity between neuroimaging variables, which impacts conclusions about the significance of effect model applicability in regression model, we checked the tolerance value of each variable predictor, that is proportion of variation in each predictor independent from the correlation between regressors (Berk 1977). The tolerance value was computed as: (1−Rj²), where Rj² is the coefficient of determination obtained by modeling the jth regressor as a linear function of the remaining independent variables. The cut-off value was set such that the variability in a predictor not related to other variables in the model was at least larger than 30%.

## Results

### Preliminary correlation analyses: BDNF levels and changes in volumetric and DTI Data

As shown in Table 2006, in the elderly subgroup BDNF levels correlated: (1) positively with normalized volume (NV) and MD of the left amygdala, and (2) negatively with bilateral hippocampus MD.

**Table 2:** Crude correlations between BDNF value and volumetric data, DTI data of 120 healthy subjects separated by age. Significant *P*-values are starred.

	Normalized volume		Mean diffusivity	
	Pearson's *r*	*P*-value	Pearson's *r*	*P*-value
*Young group*				
Left Amygdala	0.121	0.3316	−0.008	0.9461
Left Caudate Nucleus	0.130	0.2951	−0.162	0.1923
Left Hippocampus	0.003	0.9830	0.046	0.7137
Left Globus Pallidus	0.081	0.5135	−0.007	0.9538
Left Putamen	0.003	0.9840	0.070	0.5768
Left Thalamus	0.148	0.2333	0.033	0.7926
Right Amygdala	−0.142	0.2511	0.028	0.8249
Right Caudate Nucleus	0.355	0.0030^*^	0.002	0.9849
Right Hippocampus	0.051	0.6819	0.119	0.3373
Right Globus Pallidus	0.001	0.9907	−0.007	0.9577
Right Putamen	0.013	0.9183	0.042	0.7390
Right Thalamus	0.098	0.4301	0.025	0.8390
*Adult group*				
Left Amygdala	0.055	0.7648	0.199	0.2684
Left Caudate Nucleus	0.066	0.7179	−0.070	0.7021
Left Hippocampus	0.057	0.7546	0.039	0.8301
Left Globus Pallidus	−0.115	0.5265	−0.067	0.7136
Left Putamen	0.231	0.1974	0.017	0.9252
Left Thalamus	0.163	0.3675	0.063	0.7297
Right Amygdala	0.149	0.4099	0.044	0.8097
Right Caudate Nucleus	0.112	0.5383	−0.168	0.3542
Right Hippocampus	−0.070	0.7010	−0.020	0.9110
Right Globus Pallidus	−0.043	0.8133	−0.086	0.6350
Right Putamen	0.208	0.2472	−0.094	0.6041
Right Thalamus	0.239	0.1828	0.087	0.6335
*Elderly group*				
Left Amygdala	0.573	0.0072^*^	0.510	0.0202^*^
Left Caudate Nucleus	−0.054	0.8244	0.273	0.2485
Left Hippocampus	0.253	0.2866	−0.520	0.0174^*^
Left Globus Pallidus	−0.292	0.2147	0.006	0.9787
Left Putamen	−0.076	0.7535	0.066	0.7868
Left Thalamus	−0.222	0.3514	0.222	0.3511
Right Amygdala	−0.045	0.8536	0.381	0.0976
Right Caudate Nucleus	0.241	0.3110	0.350	0.1325
Right Hippocampus	0.234	0.3249	−0.469	0.0360^*^
Right Globus Pallidus	−0.363	0.1171	−0.252	0.2889
Right Putamen	−0.253	0.2859	−0.165	0.4923
Right Thalamus	−0.231	0.3324	0.106	0.6596

An ancillary result was found in the young subgroup, where the normalized volume of the right caudate nucleus positively correlated with BDNF levels.

When we further explored the relationship occurring between the amygdala and the hippocampus macromicrostructural parameters by a two-by-two approach or between each of them and age, strong positive correlations appeared, only in the elderly subject group, between: (1) NV of the left amygdala and age (r=0.543; P-value=0.012), (2) MD and NV of the left amygdala (r=0.59; P-value=0.0208); and (3) MD of the left and right hippocampus (r=0.611; P-value= 0.0034) (see Table 1995).

**Table 3:** Additional correlations in the elderly group.

Variables involved		Results	
Variable 1	Variable 2	Pearson's *r*	*P*-value
NV of the left amygdala	Age	0.543	0.0120
MD of the left amygdala	NV of the left amygdala	0.509	0.0208
MD of the right hippocampus	MD of the left hippocampus	0.611	0.0034

Finally, a number of significant anticorrelations emerged, in the whole cohort of 120 subjects, between: (1) the MD of the left hippocampus and education levels (*r *=* *−0.260; *P*-value = 0.0039), (2) the MD of the right hippocampus and education levels (*r *=* *−0.290; *P*-value = 0.0012), and (3) subject education levels and their age (*r *=* *−0.454; *P*-value <0.0001).

### Stepwise multiple regression analyses

Before running the stepwise multiple regression analyses, we computed the tolerance value for each variable significantly associated with BDNF values, in order to control for multicollinearity among variables. Such value was above the 0.30 cut-off for all variables (i.e., 0.974 for NV of left amygdala, 0.987 for MD of left amygdala, 0.482 for MD of right hippocampus, and 0.483 for MD of left hippocampus). Therefore, these variables could be included in the following multivariate regression model.

**Figure 1:**
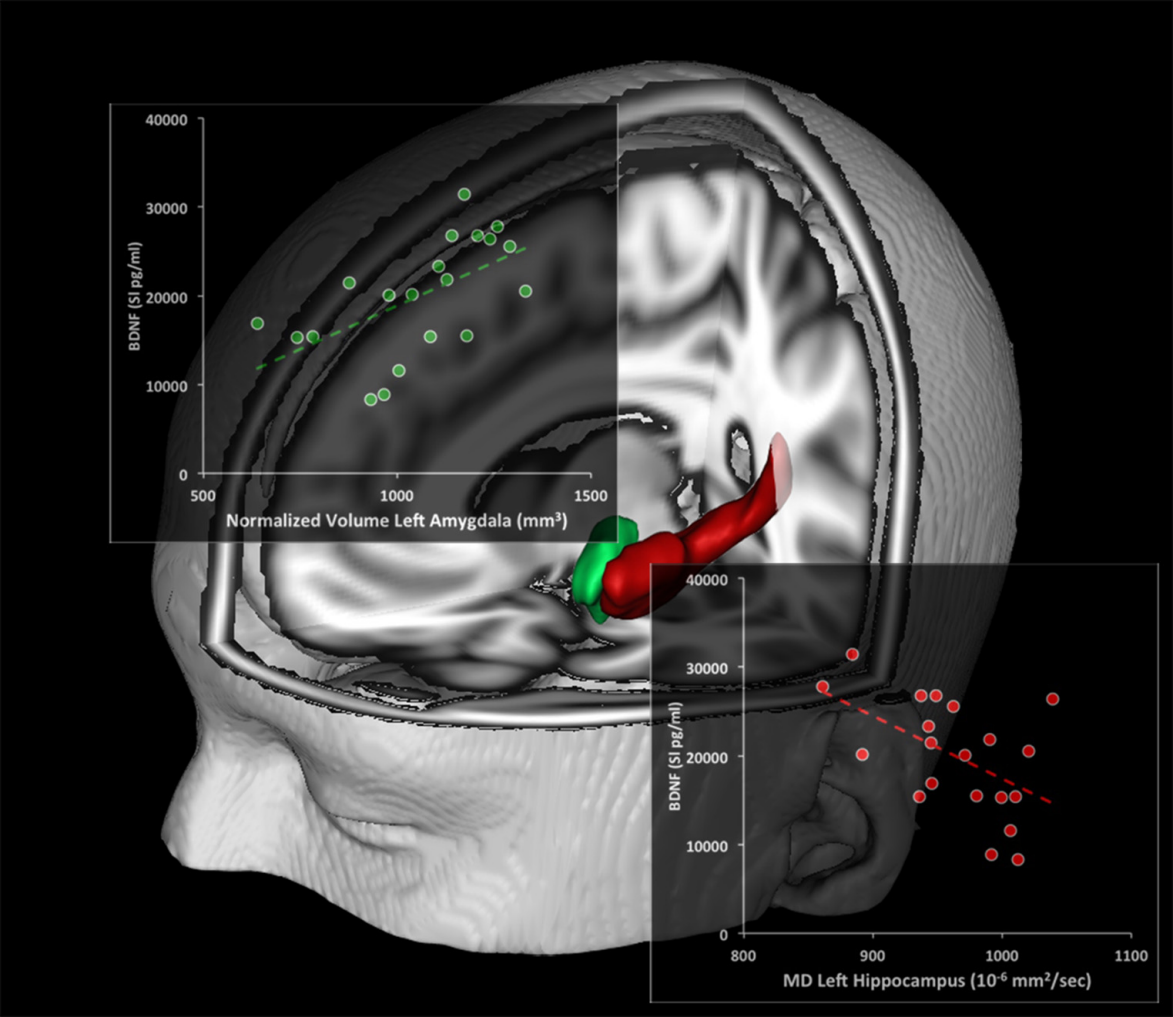
Relationship between BDNF levels and brain macro- and microstructural indices. Scatterplots show the association between BDNF levels and the left amygdala normalized volume (NV) (green) and left hippocampus mean diffusivity (MD, red). Linear fit (dotted lines) is reported.

For the initial forward stepwise regression analysis, we evaluated all the quantitative variables that emerged from the preliminary correlation analyses (NV and MD of left amygdala, MD of the left and right hippocampi) and matched these results with BDNF levels (considered as dependent variable). The only micro- and macrostructural values that entered into the regression model (as expected from the partial preliminary correlation and the statistical significance found only in the elderly group) were the NV of the left amygdala and the MD of the left hippocampus. In particular, increased BDNF values were related to increased NV of the left amygdala (beta = 0.560) and to decreased MD of the left hippocampus (beta = −0.506) (see Table 1996). No other variables reached the statistical threshold to fit the model (i.e., the remaining two factors did not act as significant predictors of BDNF levels). The equation was significant for *F* = 11.937; df = 2; *P* < 0.001; adjusted *R*^2^ = 0.535, and explained 53.5% of the overall variance of the dependent variable. Scatter plots showing positive and negative correlations between BDNF levels and: (1) NV of the left amygdala and (2) MD of the left hippocampus are shown in Figure1. These results were fully confirmed by the backward stepwise regression model in which the surviving variables were identical to those reported for the forward procedure.

**Table 4:** Forward multiple regression analysis of BDNF, using micro- and macrobrain structural quantitative variables, for 20 healthy elderly subjects. For each step of the automated procedures, beta standard coefficient and relative *P*-values for variables reaching the statistical threshold to enter into the model equation are indicated.

Variable	Forward analysis		
	Step 1	Step 2	Step 3
NV of the left amygdala	0.573 (0.008)	0.560 (0.002)	
MD of the left hippocampus	–	−0.506 (0.005)	
MD of the right hippocampus	–	–	
MD of the left amygdala	–	–	
Adjusted *R*^2^	0.291	0.535	
*F*	8.798	11.937	
Significance	<0.008	<0.001	

## Discussion

In a large cohort of healthy subjects clustered into three age groups, we investigated the relationship between levels of BDNF, a clinically relevant putative biomarker of aging, and modifications in six deep GM brain structures analyzed with quantitative MR parameters. This dataset was complemented with sociodemographic data. The main finding of the study is that, in the elderly population (age bracket: 60–76 years old), increased MD of the left hippocampus and decreased NV of the left amygdala were significantly associated with decreased BDNF peripheral levels.

The shrinkage of the hippocampal region (even if at a microstructural MD level) along with the reduced BDNF levels that we found in the elderly is in accordance with the known physiological role of the neurotrophin in maintaining hippocampal structure. Using an exploratory mediation analysis, Erickson et al. (Erickson et al. 2010) recently highlighted that BDNF modulates age-related decrease of hippocampal volume, a phenomenon that has functional consequences in terms of decay of spatial memory performances. One can speculate that the hippocampal MD increase that we have found in association with decreased BDNF levels may be an index of decreased synaptogenesis and enlargement of the extracellular space due to altered cytoarchitecture, thereby suggesting immaturity or early degeneration of the tissue (Sykova 2004; Kantarci et al. 2005).

As for the NV of the left amygdala, the positive correlation we found with BDNF serum level offers several possible interpretations. Since BDNF signaling seems particularly important in promoting amygdala-dependent LTP and can favor the acquisition and consolidation of appetitive and aversive learning events (Heldt et al. 2014), BDNF serum levels and changes in the left amygdala volume can be seen as potential risk factors for affective disorders associated with aging in the elderly (Wolf et al. 2013). Given the role of this brain region in the encoding of positive or negative information of any given event, the phenomenon can also translate in BDNF-dependent modulation of the handling of emotional content of memory encoding (Soliman et al. 2010; van Wingen et al. 2010). For example, changes in amygdala volume have been correlated quite specifically with bipolar disorders. In this regard, a recent study comparing hippocampal and amygdala volumes in two age-matched groups of elderly bipolar patients and control subjects found smaller volumes in the left amygdala of patients (Wijeratne et al. 2013). A positive association between amygdala volume and anxiety traits has also been described in healthy individuals (Barrós-Loscertales et al. 2006; van der Plas et al. 2010; Tottenham et al. 2010; Gerritsen et al. 2012).

Interestingly, we found a lateralization effect in the left hemisphere. Previous studies showed asymmetries in volume and function of the left and right hippocampus and suggested that the two hemispheres may play different but complementary roles in the modulation of memory tasks that are driven by speed (Erickson et al. 2009). Other studies that associated age-related reductions in BDNF levels with shrinkage of the hippocampi (Erickson et al. 2010, 2011) found more specific changes. These changes were occurring only in the left hippocampus, with decreased MD that negatively correlated with BDNF levels, and in the left amygdala, where volume increase positively correlated with BDNF levels. The mechanisms underlying the asymmetric shrinkage that we have observed in our elderly individuals definitely need further investigation although one can, again, speculate that neurotrophin demands are nor equally distributed between the two hippocampi or amygdala and, perhaps, these two structures of the dominant hemisphere show higher needs and suffers, prematurely, from early BDNF decreases.

Our study lends support to the assumption that MD seems to be a better parameter for investigating neurodegenerative changes occurring in the early stages or even in healthy individuals. In this context, the MD increase in the left hippocampus, which we found associated with BDNF reductions, further suggests that the parameter may be a reliable indicator of changes leading to cell loss and impaired structural integrity. Furthermore, in a recent study based on the Alzheimer's disease neuroimaging initiative (ADNI) database (adni.loni.ucla.edu), a discriminant analysis combining shape information of selected subcortical structures resulted in improved sensitivity and specificity. The authors implemented a fine characterization of atrophy patterns in the amygdala and hippocampus by integrating high-field imaging techniques and ran a Linear Discriminant Analysis (LDA) aimed at building the best LDA classifier based on shape information of seven structures and estimating the true classification rate derivable from the classification procedure. Results of the study corroborated our findings on the amygdala–hippocampal complex. Of the seven structures analyzed, the hippocampus exhibited the highest discriminative capacity; moreover, the optimal LDA originated from a combination of three structures: the hippocampus, amygdala, and lateral ventricle. When were compared individuals with mild cognitive impairment (MCI) and those with MCI who converted to AD, the authors found group surface differences primarily in these structures and detected variations in other subcortical areas. Finally, by transferring the subregions from a 0.8 mm isotropic 7.0T MR scan onto their template surfaces, the authors were able to pin down the most pronounced atrophic process to the CA1 hippocampal subregion in MCI and AD patients (Markesbery et al. 2006).

Our findings in healthy elderly individuals suggest that integrating structural information on the morphological status of several deep brain GM structures can be very helpful, in prospect, when trying to decipher the specific atrophy patterns that may take place in important and widespread neurodegenerative conditions like Alzheimer's disease (AD). For instance, a more accurate evaluation of microstructural and subregional changes can be very important and one should always keep in mind that the evaluation, as a whole, of volume modifications of any single anatomical structure often does not provide crucial information about the changes taking place at subregional levels. Still with a net result of no changes in the whole structure, some subregions may, in fact, develop atrophy that is masked by others that could have expanded. Combining BDNF levels with analysis of MD and NV can provide a more specific roadmap of the neurodegenerative pathways taken by the aging brain and AD.

We are aware that this study has some limitations. The primary one is the relatively small sample of elderly subjects, a factor that affected the strength of the correlations as well as the statistical power. Thus, results of this study warrant further investigation to definitively confirm the interplay between BDNF and lateralized amygdala–hippocampal plasticity in a larger cohort of elderly subjects. Nevertheless, we here describe an age-related association between presence of NV reduction in the left amygdala, MD increase in the left hippocampus, and reduce serum BDNF levels, thereby lending support to the idea that the neurotrophin may exert beneficial neural plasticity effect aimed at maintaining the structural integrity of these two critically important brain structures.
